# Agreement between parent and child report on parental practices regarding dietary, physical activity and sedentary behaviours: the ENERGY cross-sectional survey

**DOI:** 10.1186/1471-2458-14-918

**Published:** 2014-09-05

**Authors:** Cornelia E Rebholz, Mai JM Chinapaw, Maartje M van Stralen, Elling Bere, Bettina Bringolf, Ilse De Bourdeaudhuij, Nataša Jan, Eva Kovacs, Lea Maes, Yannis Manios, Luis Moreno, Amika S Singh, Johannes Brug, Saskia J te Velde

**Affiliations:** EMGO Institute for Health and Care Research and the Department of Public and Occupational Health, VU University Medical Center, Amsterdam, The Netherlands; Department of Public Health, Sport and Nutrition, University of Agder, Kristiansand, Norway; Department of Epidemiology and Public Health, Swiss Tropical and Public Health Institute, Basel, Switzerland; University of Basel, Basel, Switzerland; Department of Movement and Sport Sciences, Ghent University, Ghent, Belgium; Slovenian Heart Foundation, Ljubljana, Slovenia; Department of Paediatrics, Pecs University, Pecs, Hungary; Department of Public Health, Ghent University, Ghent, Belgium; Department of Nutrition and Dietetics, Harokopio University, Athens, Greece; GENUD (Growth, Exercise, Nutrition and Development) Research Group. E.U. Ciencias de la Salud, Universidad de Zaragoza, Zaragoza, Spain; EMGO Institute for Health and Care Research and the Department of Epidemiology and Biostatistics, VU University Medical Center, Amsterdam, The Netherlands

**Keywords:** Children, Parent, Parenting, Questionnaire, Inter-observer agreement, Inter-observer variability, Health behaviour, Overweight

## Abstract

**Background:**

Parents and their parenting practices play an important role in shaping their children’s environment and energy-balance related behaviours (EBRBs). Measurement of parenting practices can be parent- or child-informed, however not much is known about agreement between parent and child perspectives. This study aimed to assess agreement between parent and child reports on parental practices regarding EBRBs across different countries in Europe and to identify correlates of agreement.

**Methods:**

Within the ENERGY-project, a cross-sectional survey was conducted among 10–12 year old children and their parents in eight European countries. Both children and parents filled in a questionnaire on 14 parental practices regarding five different EBRBs (i.e. soft drink, fruit juice and breakfast consumption, sports activity and watching TV) and socio-demographic characteristics. Children’s anthropometric measurements were taken at school. We calculated percentages of agreement between children and their parents and weighted kappa statistics (for ordinal variables) per practice and country and assessed factors associated with agreement using multilevel linear regression.

**Results:**

Reports of 6425 children and their parents were available for analysis. Overall mean agreement between parent and child reports was 43% and varied little among countries. The lowest agreement was found for questions assessing joint parent–child activities, such as sports (27%; Kappa (κ) = 0.14) or watching TV (30%;κ = 0.17), and for parental allowance of the child to have soft drinks (32%;κ = 0.24) or fruit juices (32%;κ = 0.19), or to watch TV (27%;κ = 0.17). Having breakfast products available at home or having a TV in the child’s bedroom were the only practices with moderate to good agreement (>60%;κ = 0.06 and 0.77, respectively). In general, agreement was lower for boys, younger children, younger parents, parents with less than 14 years of education, single parents, parents with a higher self-reported body mass index and parents who perceived their child to be underweight.

**Conclusions:**

Parents and children perceive parental practices regarding dietary, physical activity and sedentary behaviours differently in all parts of Europe, with considerable variation across specific practices and countries. Therefore, future studies should assess both, parents and children’s view on parental practices.

**Electronic supplementary material:**

The online version of this article (doi:10.1186/1471-2458-14-918) contains supplementary material, which is available to authorized users.

## Background

The increasing prevalence of childhood overweight and obesity in the last three decades [1] has reached 26% in boys and 22% in girls in Europe [2]. There is an emerging area of research that focuses on the role of parents in influencing energy-balance related health behaviours (EBRBs) and thus weight status of their children [3–12]. A recent meta-analysis concluded that overweight prevention interventions including parent participation are more effective in maintaining a healthy body weight of child and adolescent participants than interventions without parental involvement [13]. But the role of parents in helping to control childhood overweight is multifaceted and complex [14, 15] and not all studies found an association between parenting and child weight [9]. The discrepancies found between parental practices and child’s weight status in previous studies may be explained by an interaction between general parenting style and specific parenting practices [9]. But it may also reflect the limitations of using self-report measures of weight-related behaviours and practices [15]. Unfortunately, there is no gold standard for measuring parental practices and limited information is available on psychometric properties of existing questionnaires on parenting practices [16, 17]. However, reliable and valid measures of parental practices are vital in understanding the “mechanisms” that link parental practices to health behaviour and weight status of children.

Child-reported and parent-reported measures are susceptible to recall bias and socially desirable answers. Parents may be more prone to “social desirability bias” compared to children [18]. On the other hand, children have limitations in general cognitive competencies making it more difficult for them to accurately recall past activities [18–20]. Furthermore, perceptions about parental practices may vary between children and parents [5, 18, 21–27]. Both, parents and children may therefore provide unique perspectives on their relationship and the home environment. Most studies that looked at agreement between parent and child reports regarding parental practices assessed practices related to dietary behaviour such as availability and accessibility of fruit, vegetables or soft drinks at home or the frequency of joint family meals [18, 22–25, 27]. To our knowledge only one study looked at parental practices regarding physical activity and sedentary behaviour (i.e. familial support on child’s physical activity or TV use), which had a limited number of parent–child dyads (n = 73) [21]. In general, agreement was dependent on the age of children [25, 26] and was higher when parents had a higher educational level [26]. Until now, little is known about other factors potentially influencing agreement and reliability and validity of parent and child-reported measures of parental practices.

Parental practices vary across European countries [28, 29]. In addition, the perception of parental practices varies across cultures, too [30]. We expect the frequency of parental practices as well as their perception to influence agreement between child and parent reports and thus to be different in different countries. Therefore, using the dataset of the ENERGY cross-sectional survey, including eight European countries, this study aims i) to assess agreement between child and parent report of parental practices regarding EBRB across Europe and ii) to identify correlates of agreement.

## Methods

### Study population

The EuropeaN Energy balance Research to prevent excessive weight Gain among Youth (ENERGY) project aimed to develop and evaluate a theory-informed and evidence based multi-component school-based and family involved intervention program (http://www.projectenergy.eu). [31]. In 2010 a school-based, parent-involved cross sectional study was carried out as part of the ENERGY project in eight European countries: Belgium, Greece, Hungary, Netherlands, Norway, Slovenia, Spain and Switzerland [32]. This cross-sectional study aimed to provide information regarding overweight, obesity and waist circumference in representative samples in these countries, as well as information regarding EBRBs and their personal, social and physical environmental correlates. A minimum sample of 1000 children aged 10 to 12 years per country and one parent for each child was required to detect a between country difference of 5% in obesity prevalence as statistically significant with a power of 90%. The sampling of schools was random, multi-staged and stratified by degree of urbanization [32]. In total, 202 schools were recruited and 7915 children and 6463 parents/caregivers filled in a questionnaire. Response rates of the children were in general high (≥64%) except for Hungary (33%), Norway (45%) and Spain (43%) [2]. The response rate of parents was also high (>60%) except for the Netherlands (41%). The survey in Switzerland was conducted later than in other participating countries and its results have been published elsewhere [33]. For the current analysis we excluded data from 38 caregivers other than mother or father who filled in the parental questionnaire, resulting in 6425 parent–child dyads.

The project adhered to the Helsinki Declaration and the conventions of the Council of Europe on human rights and biomedicine. Ethical clearance was obtained from the relevant ethical committees and ministries in all participating countries (a detailed list including names and affiliations is provided in Additional file 1: Table S5). In addition, research permission was obtained from local school authorities if necessary. Passive informed consent from the parents was allowed in the Netherlands [32]. In all remaining countries parents were asked for written consent for their child’s and their own participation.

### Measurements

The selection of EBRBs and correlates assessed in the questionnaires was based on literature reviews and secondary data analyses conducted within ENERGY [34, 35]. Both child and parent questionnaires were developed using items from validated European and country-specific questionnaires and if needed new items were added. Once created, the questionnaires were translated back and forward into the language of each participating country and were pretested in small samples in all participating countries (available in all languages at http://www.projectenergy.eu).

#### Parental practices

Parental practices are defined as specific behaviours and factors related to behaviour management (e.g. limit setting, availability) and social cognitions (encouragement, norms) for the following EBRBs: soft drink consumption, fruit juice intake, having breakfast, physical activity/sports and watching TV (Additional file 2: Table S1). These practices include availability of products at home, accessibility to products by allowing the child to have them, encouraging the child to engage in certain behaviours and performing behaviours together. All parental practices were assessed by single items using a 5-point Likert scale except for availability of a television (TV) set in the child’s own bedroom, which was a yes/no question. These items were informed by the Pro Children Questionnaire [36] and the ENDORSE study questionnaire [37]. Parental practices as reported in the child questionnaire showed in general good to excellent test-retest reliability (ICCs > 0.60-1.00) while construct validity for most parental practices, however, was moderate to poor (ICCs < 0.60, percentage agreement with interview <60%) (Additional file 2: Table S1) [38]. Similarly, the ENERGY-parent questionnaire showed a good to excellent test-retest reliability for parental practices while construct validity was moderate for four and poor for two out of thirteen parental practices (Additional file 2: Table S1) [39].

#### Potential correlates of agreement

In each country body height, weight and waist circumference of the children were measured by two trained research assistants according to a standardized protocol [32]. Their weight status was classified into underweight using cut-off values as defined by Cole et al. [40], normal weight and overweight using IOTF standard definitions [41]. Besides engagement in different EBRBs, i.e. soft drink and fruit juice consumption, having breakfast, taking part in physical activities/sports and watching TV, children were asked to report their date of birth, gender, the language they most often speak at home and with whom they were living with (parents, either mother or father only, or other adults). Parents reported who was filling in the questionnaire (mother or father), socio-demographic information, their weight and height, and the years of completed school education. Answer categories were: a) less than 7 years, b) 7–9 years, c) 10–11 years, d) 12–13 years and e) 14 years or more. Based on the distribution of the data, parental education was categorized as being high (at least one parent had more than 14 years of education) or low (both parents less than 14 years of education), which approximately distinguishes families with at least one caregiver who has completed medium or higher vocational, college or university training from other families [2, 42]. Finally, parents reported what they thought about the weight status of their child: normal, a bit or way too much or a bit or way too little.

### Statistical analysis

Descriptive statistics were used to characterize parent–child dyads available for analysis. Factors from the child questionnaire that were associated with non-response of parents were identified using multilevel logistic regression including gender of the child, age and BMI, the living situation and whether the family spoke the national language at home, overall (level 1 country, level 2 schools) and for each country (level 1 schools) separately. To assess overall agreement between child and parent reports on parental practices we created an agreement score of all 14 practices ranging from 0 (no agreement at all) to 100 (agreement in all practices). An average agreement score was calculated for parent–child dyads that answered at least 10 out of 14 practices (6358 out of 6425; 99%). We calculated percentage agreement between child and parent report for each parental practice in total and for each of the participating countries separately. The established criteria for percentage agreement were “good to excellent” (≥75%), “moderate” (60%-74%) and “poor” (<60%) [38, 39]. Country specific percentage agreement was explicitly mentioned in the text if agreement between countries differed by more than 10%, a cut off arbitrarily chosen, but roughly distinguishing relevant from small irrelevant differences, to facilitate reading of the large Table 1. Furthermore we looked at the direction of disagreement (i.e. did children on average score higher or lower than parents) by calculating the mean difference in scores of parent and child reports. To report the size of disagreement we then calculated the root of the squared difference for each practice. In addition, we calculated weighted Cohen’s kappa coefficients to assess the agreement between child and parent report. Weighted Cohen’s kappa takes into account the degree of disagreement, i.e. that the disagreement is greater if categories are further apart (e.g. ‘completely agree’ vs. ‘completely disagree’ rather than ‘completely agree vs. ‘agree somewhat’). We assigned a linear set of weights. In case of items with 5 answer categories, this means that weights were 1, 0.75, 0.50, 0.25 and 0 when there is a difference of 0 (=total agreement) or 1, 2, 3, and 4 categories respectively. Cohen’s kappa coefficients were defined as following: <0.0 poor, 0.00-0.20 slight, 0.21- 0.40 fair, 0.41-0.60 moderate, 0.61-0.80 substantial and 0.81-1.00 almost perfect agreement [43]. We repeated the agreement analysis recoding the 5 answer categories into a 3-point scale, i.e. always or often versus sometimes versus not often or never.

**Table 1:** **Agreement between child and parent report on parental practices regarding five different energy-balance related behaviours**

	Belgium				Greece				Hungary				Netherlands				Norway				Slovenia				Spain				Switzerland				Total				Total (3-point scale)	
	κ	%*	Diff ^†^	§	κ	%*	Diff ^†^	§	κ	%*	Diff ^†^	§	κ	%*	Diff ^†^	§	κ	%*	Diff ^†^	§	κ	%*	Diff ^†^	§	κ	%*	Diff ^†^	§	κ	%*	Diff ^†^	§	κ	%*	Diff ^†^	§	κ	%*
**Parental practices**																																						
*Soft drink consumption*																																						
“If I ask my parents for a fizzy drink of fruit squash, I get one”/“If my child asks for soft drinks, I will give it to him/her”	0.30	42.3	0.71	^+^	0.15	40.8	0.79	^+^	0.19	33.7	0.95	^+^	0.15	39.1	0.81	^+^	0.15	47.7	0.61		0.19	34.6	0.89		0.17	40.2	0.76		0.16	35.5	0.81	^+^	0.21	39.2	0.80	^+^	0.23	46.0
“I am allowed to take fizzy drinks or fruit squash whenever I want”/“My child is allowed to take soft drinks whenever (s)he wants”	0.36	35.5	0.94	^+^	0.18	35.0	0.91	^+^	0.20	27.9	1.22	^+^	0.17	25.6	1.18	^+^	0.18	36.4	0.88	^+^	0.22	28.4	1.15	^+^	0.18	33.4	0.92	^+^	0.18	27.0	1.13	^+^	0.24	31.7	1.03	^+^	0.26	49.6
“Are there usually fizzy drinks or fruit squash at your home?”/“There are soft drinks available at home for my child”	0.23	34.7	0.98	^+^	0.22	34.6	0.94	^+^	0.27	32.3	1.01	^+^	0.16	30.6	1.05	^+^	0.15	33.0	0.95		0.22	34.0	0.99	^-^	0.22	33.9	1.04		0.32	38.4	0.86	^+^	0.27	33.9	0.98	^+^	0.29	47.8
*Fruit juice consumption*																																						
“I am allowed to take fruit juices whenever I want”/“My child is allowed to take fruit juices whenever (s)he wants”	0.22	32.7	1.17	^+^	0.16	35.0	1.01		0.11	29.4	1.17	^-^	0.15	26.1	1.18	^+^	0.25	31.2	0.99	^+^	0.20	36.5	1.02		0.18	31.9	1.10		0.19	24.1	1.37	^+^	0.19	31.7	1.12	^+^	0.20	50.7
“Are there usually fruit juices in your home?”/“There are fruit juices available at home for my child”	0.27	39.2	0.82	^-^	0.20	43.9	0.81	^-^	0.20	34.9	0.95		0.20	37.8	0.91		0.43	44.6	0.67		0.21	34.6	0.95		0.25	43.5	0.82	^-^	0.33	37.9	0.86	^+^	0.28	39.7	0.85		0.29	60.5
*Having breakfast*																																						
“My parents encourage me to have breakfast”/“I encourage my child to have breakfast”	0.04	42.7	1.23	^-^	0.10	43.0	0.99	^-^	0.10	63.0	0.60	^-^	0.02	28.1	1.75	^-^	0.02	35.1	1.30	^-^	0.12	54.9	0.83	^-^	0.05	48.2	1.28	^-^	0.02	28.4	1.48	^-^	0.06	45.2	1.11	^-^	0.06	63.8
Are there usually breakfast products (e.g. milk, cereal, bread) at your home?“/”There are breakfast products (e.g. milk, cereal, bread) available at home for my child“	-0.01	71.1	0.34	^-^	0.15	74.0	0.33	^-^	0.09	73.0	0.34	^-^	0.03	69.2	0.36	^-^	0.03	71.4	0.32	^-^	0.02	59.6	0.54	^-^	0.01	80.6	0.22	^+^	0.07	66.1	0.39	^-^	0.06	70.9	0.36	^-^	0.05	90.7
”How often do you eat breakfast with your parents?“/”How often do you and/or your spouse/partner have breakfast together with your child?“	0.42	45.3	0.86	^+^	0.24	34.2	1.06	^+^	0.28	37.2	0.93		0.30	44.6	0.82	^-^	0.31	40.9	0.85	^-^	0.27	41.7	0.90	^+^	0.28	38.2	1.09		0.41	47.1	0.81	^-^	0.35	40.3	0.94		0.35	50.0
*Physical activity/sports*																																						
”My parents encourage me to be physically active/do sports“/”I encourage my child to take part in physical activity/sports“	0.08	44.0	0.92	^-^	0.16	41.8	0.74		0.17	58.6	0.57	^+^	0.05	34.1	1.11	^-^	0.11	44.7	0.82	^-^	0.13	57.3	0.64	^+^	0.10	46.2	0.81	^-^	0.06	34.5	1.08	^-^	0.12	46.8	0.80		0.09	69.3
”How often do you take part in physical activity/do sports with your parents?“/”How often do you and/or your spouse/partner participate in physical activity/sports together with your child (e.g. Play games outside, ride bikes, walk, play sports together)?“	0.13	28.9	0.81		0.14	29.6	0.87	^+^	0.11	26.8	0.99	^+^	0.13	25.6	1.00	^-^	0.20	31.9	0.76	^-^	0.10	27.9	0.95		0.14	23.3	0.96	^+^	0.08	19.6	0.91	^-^	0.14	27.1	0.90		0.12	59.0
*Watching TV*																																						
”My parents allow me to watch television whenever I want“/”My child is allowed to watch TV/video/dvd whenever (s)he wants“	0.22	28.3	1.11	^+^	0.14	30.4	1.07		0.20	26.5	1.23	^+^	0.23	24.3	1.07	^+^	0.11	25.7	1.12	^-^	0.11	21.4	1.31	^+^	0.11	25.4	1.11		0.22	34.3	0.91	^+^	0.17	26.8	1.13	^+^	0.19	43.0
”If I ask my parents to watch television, I can do so“/”If my child asks if (s)he is allowed to watch TV/video/dvd, I will allow it“	0.18	42.6	0.65	^+^	0.17	38.5	0.76		0.14	34.7	0.86	^+^	0.32	50.4	0.55		0.21	47.3	0.59		0.16	39.7	0.74	^+^	0.11	40.8	0.70		0.14	44.8	0.64		0.21	41.4	0.70	^+^	0.23	52.2
”Do you have a television in your own bedroom?“/”TV/video/DVD is available in my child“s room?”^ǁǁ^	0.77	89.8			0.75	87.1			0.70	85.0			0.77	87.7			0.80	89.8			0.68	85.9			0.79	78.8			0.72	89.1			0.77^‡^	86.3			0.77	86.3
“How often do you watch television with your parents?”/“How often do you (one parent/spouse/partner or both) watch television together with your child?”	0.26	28.9	1.09	^+^	0.20	31.1	1.10		0.24	26.3	1.15	^+^	0.28	31.3	1.03	^-^	0.20	37.6	0.87	^-^	0.21	29.2	1.05	^+^	0.24	27.4	1.13	^-^	0.27	31.8	0.96	^+^	0.25	30.2	1.06		0.27	46.7

*Abbreviations*: *κ* weighted Kappa statistics.

*Percentage of agreement between child and parent reports.

^†^Mean of square root of squared difference between child and parent report.

§Children score higher (+) or lower (-) than their parents, mean difference > ± 0.1.

^ǁǁ^Answer categories included Yes/No.

^‡^Unweighted Kappa.

Factors associated with the agreement score were assessed using univariate multilevel linear regression for the combined dataset (level 1: country, level 2: school) as well as for each country separately (level 1: school). The assumption of normally distributed residuals was checked and met. Next, a multilevel linear regression was conducted including all factors that were found to be significantly associated with agreement in univariate analysis. Stata version 11 (StataCorp, College Station, TX, USA) was used for all analyses. P-value < 0.05 was considered as statistically significant.

## Results

### Characteristics of the study population

Table 2 presents the characteristic of the children and their parents. Of the children 23% were overweight or obese and 8% underweight. Country specific results are presented in supplemental tables. Table 2 also shows the results of the non-response analyse, showing that parents of boys, non-native speakers of the national language and parents who were single mothers or fathers and parents of children with higher BMI were more likely to be non-responders. Similar associations were observed across countries with a few exceptions that are shown in Additional file 3: Table S2.

**Table 2:** **Characteristics of parent–child dyads and factors associated with non-response of parent**

		Participants		Non-responders*					
		N	%	N	%	OR ^†^	95% CI		
**Child - parent dyads**		6′425		1′452					
**Child**									
Gender	Girls	3′423	53	675	47	1.00			
	Boys	3′002	47	777	54	0.73	0.63	-	0.84
Age (years)	10 < 11	1,418	22	226	16	1.00			
	11 < 12	2,859	45	530	37	0.96	0.78	-	1.18
	12 < 13	1,933	30	523	36	0.69	0.55	-	0.85
	13 < 14	205	3	81	6	0.40	0.27	-	0.58
National lan-guage at home	No	422	7	182	13	1.00			
	Yes	5′951	93	1′199	83	2.45	1.92	-	3.12
Living with	Both parents	5′335	83	1′132	78	1.00			
	Mother or father	581	9	186	13	0.65	0.52	-	0.82
	Other adults	466	7	65	5	0.90	0.66	-	1.22
Siblings	No	988	15	176	12	1.00			
	Yes	5′384	84	1′203	83	0.87	0.71	-	1.07
Weight status	Normal	4′354	68	955	66	n.a.			
	Overweight^‡^	1′470	23	295	20				
	Underweight^§^	506	8	108	7				
		**Mean**	**SD**	**Mean**	**SD**				
BMI of child	kg/m2	19.01	3.29	18.99	3.38	0.97	0.95	-	0.99
**Parent**		**N**	**%**						
Gender	Mother	5′297	82						
	Father	1′107	17						
Education	< 14 years	2′800	44						
	≥ 14 years	3′560	55						
		**Mean**	**SD**						
Age of parent	years	41.34	5.20						
BMI of parent	kg/m^2^	24.50	4.25						

*Abbreviations*: *BMI* Body mass index, *CI* Confidence Interval, *OR* Odds ratio.

*Parents who did not return a questionnaire.

^†^Multilevel (country, school) logistic regression analyses with response as outcome variable including all child characteristics listed (including BMI as a continuous variable).

^‡^Using IOTF standard definition [41].

^§^Using cut-off value as defined by Cole et al., BMJ, 2007 [40].

Percentages do not always add up to 100% due to missing values and rounding.

### Parental practices

Considerable variation in parental practices was observed across countries (Additional file 4: Table S3). For example 47% of children in Greece reported to never or less than once a week have breakfast with their parents while this was true for 13% of children in the Netherlands. In Swiss children 11% reported that their parents would allow them to often or always watch TV if they asked for it as compared to 46% in Hungarian children. Dutch and Swiss children (40% and 41%, respectively) reported the lowest parental encouragement to be physically active or to do sports; Slovenian children the highest encouragement (83%). Children in Belgium and the Netherlands more often reported soft drinks to be available at home than children from other countries.

### Overall agreement by country

**Figure 1:**
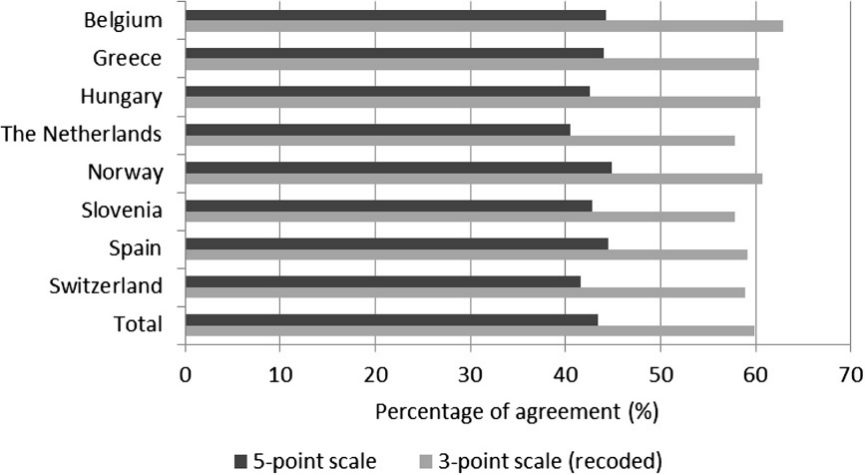
**Average agreement score (%) between parent and child reports on parental practices, overall and by country.** An average agreement score was calculated for parent–child dyads that answered at least 10 out of 14 practices; using exact answers and recoded answers (i.e. the 5 answer categories were recoded into a 3-point scale, e.g. always or often versus sometimes versus not often or never).

The mean total agreement score, i.e. including all parental practices, between child and parent reports was 43% (Standard deviation = 14; Figure 1). Mean total agreement score was similar in all countries ranging from 41% in the Netherlands to 45% in Norway and Spain.

### Agreement by practice

Agreement between child and parent reports varied by parental practice.

#### Co-participation

In general, parental practices that included joint parent–child activities such as eating breakfast together (Kappa (κ) = 0.35;40%), engaging in physical activity or doing sports (κ = 0.14;27%) and watching TV (κ = 0.25;30%) yielded poorer agreement between child and parent reports than other practices (Table 1). Mean differences using the 5-point Likert scale were 0.94, 0.90 and 1.06 points, respectively. There was no general trend of children or parents scoring higher. Agreement on having breakfast together ranged from fair (κ = 0.24;34%) in Greece to moderate in Belgium and Switzerland (κ = 0.42;45% and κ = 0.41;47%, respectively).

#### Rules

Slight to fair agreement was found for the parental practices allowing soft drink (κ = 0.24;32%) and fruit juice (κ = 0.19;32%) consumption to the child and allowing the child to watch TV (κ = 0.17;27%) whenever he/she wants. For all parental practices in this domain children scored in general higher than their parents, meaning that they reported more often to be allowed to have soft drinks, fruit juice and watch TV than the parents reported they allowed them to. Agreement in allowed watching TV varied from slight (κ =0.11,;21%) in Slovenia to fair (κ = 0.22,;34%) in Switzerland.

#### Encouragement

Average agreement on parental encouragement for having breakfast was slight (κ = 0.06;45%) and ranged from fair (κ = 0.02,;35%) in Norway to substantial (κ = 0.10;63%) in Hungary. In all countries, children reported less often than parents parental encouragement for having breakfast. Agreement on parental encouragement for taking part in physical activity was also slight (κ = 0.12;47%) and the direction of agreement varied across countries. Agreement on parental encouragement for physical activity was poorest in the Netherlands (κ = 0.05;34%) and best, but still slight, in Hungary (κ = 0.17;59%).

#### Availability

Agreement between parent and child on availability of products in their homes was slight regarding breakfast products (κ = 0.06;71%) and fair regarding soft drinks (κ = 0.27;34%) and fruit juice (κ = 0.28;40%). Compared to their parents, children more often reported soft drinks to be available at home. This was not the case for fruit juice availability.

### Sensitivity analyses

Using the recoded 3-point scales resulted in higher agreement between parent and child responses. For all parental practices the mean total agreement score reached 60% (SD = 15) and was similar in all countries ranging from 58% in the Netherlands and Slovenia to 63% in Belgium (Figure 1). Furthermore, agreement between parent and child report was higher for joint sport activities (59%), and to some extent for having breakfast (50%) and watching TV (47%) together when using the recoded 3-point scale compared to when using the original 5-point scale (Table 1).

### Factors associated with agreement

In multilevel univariate analyses agreement was lower for boys, for younger children and for younger parents and parents with less years of education (Additional file 5: Table S4). In addition, agreement between child and parent report was lower for overweight children and for children whose parents perceived them to be either over- or underweight.

Our results indicate that agreement was significantly lower in boys (regression coefficient “b” = -1.84; 95% Confidence interval “CI” = -2.59 to -1.10; p-value < 0.0001) (Table 3). Further, agreement improved with increasing age of the child (b = 1.06; 95% CI -0.04 to 2.16 and 2.41; 95% CI 0.12 to 4.71 for age 12 and 13 years, respectively as compared to age 10 years; p-value = 0.013) and of the parent (b = 0.08 per year; 95% CI 0.00 to 0.15; p-value = 0.041). More than 14 years of parental education was associated with better agreement between child and parent reports as compared to fewer years of education (b = 2.08; 95% CI 1.28 to 2.88; p-value < 0.0001). Lower agreement was observed for single parents as compared to two parent households (b = -1.31; 95% CI -2.62 to -0.00; p-value = 0.050). Parents with a higher self-reported BMI (b = -0.11 per kg/m^2^; 95% CI -0.20 to -0.02; p-value = 0.013) and parents who perceived their child to be underweight showed lower agreement scores (b = -1.11; 95% CI -2.28 to 0.07; p-value = 0.039). In general, the directions of these associations were observed in all countries, although not always statistically significant. In Belgium, parents who perceived their child to be overweight agreed more with their child on parental practices (b = 3.81; 95% CI 0.39 to 7.22; p-value = 0.029). The direction of this association varied across countries. Furthermore, speaking the national language at home increased agreement between children and parents in Belgium and Slovenia (b = 4.35; 95% CI -0.05 to 8.75; p-value = 0.052 and b = 4.28; 95% CI 0.38 to 8.18; p-value = 0.032, respectively).

**Table 3:** **Factors associated with agreement between child and parent report (agreement score), overall and by country**

		Total			Belgium			Greece			Hungary			Netherlands			Norway			Slovenia			Spain			Switzerland		
		b*	95% CI		b ^†^	95% CI		b ^†^	95% CI		b ^†^	95% CI		b ^†^	95% CI		b ^†^	95% CI		b ^†^	95% CI		b†	95%CI		b†	95% CI	
*Child characteristics*																												
Gender																												
	Boy	**-1.84**	**-2.59**	**-1.10**	-2.03	-4.20	0.13	-1.78	-3.69	0.12	-1.22	-3.21	0.76	-0.77	-3.63	2.09	**-3.49**	**-5.48**	**-1.49**	-1.30	-3.26	0.66	-1.77	-3.71	0.16	-1.70	-4.26	0.86
Age	10 years																											
	11 years	0.20	-0.77	1.18	-0.13	-2.73	2.47	-1.92	-4.05	0.20	1.10	-10.69	12.88	-0.63	-4.57	3.30	**0.68**	**1.71**	**2.36**	1.44	-0.79	3.68	1.16	-1.11	3.43	-1.97	-5.16	1.23
	12 years	1.06	-0.04	2.16	-0.38	-3.40	2.64	0.43	-2.36	3.23	1.17	-10.57	12.91	1.63	-2.56	5.82	**1.42**	**1.70**	**2.80**	0.16	-2.56	2.88	**4.16**	**1.39**	**6.93**	-0.08	-3.67	3.51
	13 years	**2.41**	**0.12**	**4.71**	4.88	-5.82	15.57	**15.02**	**2.15**	**27.89**	3.00	-9.13	15.13	-2.37	-15.83	11.08	**1.75**	**2.30**	**2.72**	-14.30	-35.19	6.59	1.94	-14.40	18.29	-3.43	-10.58	3.72
Weight status																												
	Normal																											
	Overweight	-0.73	-1.92	0.46	-1.28	-4.95	2.40	-0.14	-2.77	2.49	-1.65	-4.79	1.49	-1.61	-6.73	3.50	0.09	-3.54	3.72	0.39	-2.67	3.46	0.95	-2.04	3.93	-2.98	-8.12	2.16
	Underweight	0.06	-1.43	1.54	0.94	-2.69	4.58	0.49	-5.15	6.13	2.24	-1.50	5.99	3.12	-1.80	8.03	-3.02	-7.01	0.97	-0.09	-3.99	3.82	-2.31	-7.12	2.51	0.77	-3.49	5.03
*Parental characteristics*																												
Age	per year	**0.08**	**0.00**	**0.15**	0.14	-0.10	0.37	0.09	-0.09	0.26	-0.01	-0.20	0.19	0.24	-0.08	0.56	0.13	-0.07	0.33	0.06	-0.15	0.26	0.12	-0.10	0.35	0.02	-0.22	0.25
BMI	per kg/m2	**-0.11**	**-0.20**	**-0.02**	-0.07	-0.33	0.18	0.05	-0.16	0.26	-0.04	-0.25	0.18	**-0.36**	**-0.66**	**-0.06**	-0.15	-0.42	0.11	**-0.31**	**-0.55**	**-0.07**	-0.22	-0.52	0.07	0.06	-0.27	0.39
Education																												
	<14 yrs																											
	≥ 14 yrs	**2.08**	**1.28**	**2.88**	1.04	-1.62	3.70	**2.69**	**0.73**	**4.66**	1.18	-0.89	3.26	1.35	-1.90	4.61	**3.90**	**1.78**	**6.03**	**3.37**	**1.31**	**5.43**	0.85	0.61	1.42	0.61	-2.18	3.40
Employment																												
	Yes	-0.37	-1.45	0.71	0.44	-3.48	4.37	-1.40	-3.85	1.05	-0.13	-2.87	2.61	3.11	-0.55	6.76	-0.48	-4.59	3.62	-0.14	-3.90	3.62	-2.42	-4.91	0.07	1.88	-1.22	4.97
Perception of the child’s weight																												
	Normal																											
	Overweight	-0.12	-1.31	1.07	**3.81**	**0.39**	**7.22**	0.29	-2.33	2.90	-0.96	-4.13	2.21	3.41	-1.28	8.11	-2.39	-6.30	1.53	-1.97	-5.01	1.08	-2.81	2.78	2.51	2.78	-2.14	7.70
	Underweight	**-1.35**	**-2.63**	**-0.07**	-1.46	-5.22	2.30	0.43	-3.26	4.12	**-4.90**	**-8.31**	**-1.48**	-3.05	-7.99	1.89	-3.14	-6.69	0.41	-0.59	-3.98	2.81	-0.41	-3.63	2.81	2.03	-2.17	6.23
*Family characteristics*																												
Native language at home																												
	Yes	0.40	-1.19	2.00	4.35	-0.05	8.75	-2.14	-6.02	1.74	0.55	-5.99	7.09	-5.23	-11.95	1.49	1.90	-3.94	7.74	**4.28**	**0.38**	**8.18**	-5.57	-12.31	1.17	0.00	-3.20	3.19
Living with																												
	Both parents																											
	Mother or Father	**-1.31**	**-2.62**	**0.00**	-0.64	-4.65	3.38	-2.39	-5.96	1.18	-0.40	-3.38	2.57	-2.68	-7.17	1.81	1.53	-1.84	4.91	**-4.31**	**-8.18**	**-0.43**	-2.41	-6.61	1.80	0.04	-3.76	3.84
	Other	-1.22	-2.67	0.22	**-10.52**	**-18.32**	**-2.72**	-1.28	-4.15	1.60	-2.07	-5.28	1.13	-12.23	-38.65	14.18	2.19	-5.46	9.83	-1.40	-4.43	1.62	1.64	-1.84	5.13	-5.85	-14.14	2.44

*Abbreviations*: *CI* Confidence Interval, *BMI* Body mass index.

Significant associations are highlighted in bold.

## Discussion

The aim of the current study was to assess agreement between parent and child reports on specific parenting practices. We did not aim to decide on what perspective is most valid as no golden standard exists to assess parental practices. Based on the data of the ENERGY cross-sectional survey, we found poor agreement between child and parent reports on parental practices regarding dietary, physical activity and sedentary behaviours in eight European countries; ranging from 41% in the Netherlands to 45% in Norway and Spain. However, agreement varied substantially across different parental practices and was lowest for practices including joint child–parent activities, i.e. engaging jointly in physical activity (27%) and watching television together (30%), as well as for allowing taking soft drinks (32%), fruit juice (32%) or watching TV (27%). Overall agreement was poorer for boys, younger children, younger parents, parents with fewer years of education, single parents and parents with a higher BMI.

### Agreement between child and parent report

In general we found that parents and children responded differently to questions assessing parenting practices. This disagreement may be explained by several factors.

Firstly, parents and children may have different perspectives on their relationship and behaviours [5, 10, 21, 27]. Despite differences in methodologies and questionnaires, considerable disagreement between parents and children has been reported before. A study by van Assema et al. [27] reported low agreement for perceived availability and accessibility of snack, fruit and breakfast products. Barr-Anderson et al. found low levels of agreement between child and parent reports regarding family support for physical activity and parental limitations on child’s TV use (ranging from 25%-42%) and good agreement (≈70%) if child and parent responses were allowed to differ by one point in the 4-point scale [21]. These results suggest that future studies should take both perspectives into account.

A second explanation for the disagreement may be that parents are more likely to give socially desirable answers [18]. However, we did not find that parents consistently reported more favourable scores. On the contrary Barr-Anderson et al. reported that in their study children perceived their parents to be more supportive than parents rated themselves suggesting that parents may have viewed their small actions of support (encouraging words, providing rides) as part of their parental duties [21]. Similarly, joint child–parent activities may be perceived differently by children and parents. If social desirability was at stake we expect parents to report joint sports activities more frequent and watching TV together less frequent than their children. However, such a pattern was only observed in Switzerland. On the other hand, parents may also perceive watching TV together, i.e. social co-viewing, as a favourable behaviour as in this way they can better monitor what they children watch counteract undesirable effects of television programs and commercials [44]. Likewise, we do not know whether children in this study more often reported being allowed to consume soft drinks or fruit juice and to watch TV than their parents due to different perceptions or rather due to socially desirable answers of their parents. Parents may be aware of potential unfavourable effects of excessive soft drink consumption and TV viewing and realize that they should limit these behaviours and therefore provide a desirable answer. Children, on the other hand, may have given more honest answers, resulting in disagreement with their parent’s answer especially in children who drink more soft drinks and watch more TV.

Thirdly, a methodological explanation for low agreement, especially for some items, can be the low test-rest reliability and validity. Practices with low agreement i.e. engaging in physical activity or doing sports together, watching TV together, allowing the child to take soft drinks, fruit juices or watch TV also had lower test-retest reliability and validity (Additional file 2: Table S1) [38, 39], especially in the child questionnaire. A possible explanation is that these practices vary more on a daily basis and are thus less stable and clear. In addition, questions on joint parent–child activities as well as on availability of a TV in the bedroom were framed slightly different in the parental and child questionnaire, which may also partly explain the lower agreement found for these parental practices as compared to others. Another explanation may be the limitations in memory capacities and cognitive competencies in children [18–20].

Finally, the sensitivity analyses with the recoded 3-point score for the parenting practices showed that this recoding strongly influenced the agreement between child and parent reports and reached moderate levels (60%). We assessed this because many studies (including studies based on the ENERGY data) recode original 5-point scales into 3-point scales for methodological reasons (e.g. skewness, empty cells). These 5-catogory variables cannot be used as continuous variables in analyses and are usually recoded into a lower number of categories to make analyses and interpretation easier. As the majority of parents and children tended to choose the same answer category, remaining cells in a 5 × 5 table of parent and child scores on parental practices were left with very low numbers, resulting in low kappa values. Kappa is affected by the prevalence of the finding under consideration and very low values of kappa may not necessarily reflect low rates of overall agreement [45]. Therefore we decided to present both, kappa values and percentage of agreement. This problem can be avoided by using two separate indexes of proportionate agreement: i.e. the observed proportion of positive agreement and the observed proportion of negative agreement. These two indexes are analogous to sensitivity and specificity for concordance in a diagnostic marker test [46].

### Correlates of agreement

Our findings show that agreement increases with children’s age. Cognitive competencies of children develop with age, i.e. the information process mechanisms that enable them to attend to, select, represent and act on information as well as working memory functions that support the mental representation of information [19]. The lower agreement found for boys and their parents in our study might be explained by an earlier start of psychosocial maturation in girls compared to boys [47]. A low capability of self-reflection and self-concept in adolescents have been associated with poor communication with their parents [48]. Likewise, in two previous studies, girls agreed more with their parents on the availability of specific fruits at home [49] and on the frequency of family meals than boys [24]. It may also be that girls are more inclined to give socially desirable answers than boys and therefore their answers may be more in agreement with their parents [50]. Agreement was higher in older parents. Older parents may have more routine as well as more clearly defined parental practices. In addition, the child who has older parents is more likely to have an older sibling who might remind him of the parental practices in their family. The observed higher agreement between parent and child reports with higher level of parental education corresponds to findings of previous studies on agreement between parent and child reports regarding parental practices [26, 51]. A higher BMI of parents was associated with lower agreement in our study. Parents with a higher BMI may use different parental practices and may be more prone to giving social desirable answers. In an observational study parents with a higher BMI applied more controlling feeding practices and maternal BMI was related to greater reported than observed restriction of their children’s intake [52]. A significant proportion of parents tend to underestimate their child’s weight [53] especially if children and parents themselves are overweight [54], which might be partly explained by the parental desire to not be judged as a bad parent. However, more research is needed to understand the mechanisms of how parental perception of weight status in their child influences agreement between child and parent report on parental practices. Our data suggests the existence of underlying cultural factors, which we have not assessed.

### Variation by country

Agreement between child and parent reports was similar across countries despite a large variation in parental practices. This indicates that such agreement is rather stable and robust, and influenced by other factors than cultural differences or country differences in parenting practices between the included countries. Similarly, in a study comparing parent-reported and adolescent self-reported problems in 25 societies found that parent-adolescent item agreement varied widely across dyads but mean dyadic agreement was modest in every society [55].

### Strengths

As part of the ENERGY project [31] eight European countries participated in the cross-sectional survey [32] resulting in a large dataset reflecting different cultures. This is important since parental practices and the perception of these practices are likely to vary between cultures [30]. A set of variables potentially influencing agreement between child and parent reports, such as objectively assessed BMI of children and the parental perception of their child’s weight status, were available for analysis. Test-retest reliability was good for both parent and child questionnaires and construct validity was good and moderate to good for most but parental practices items in the parent and child questionnaire, respectively [38, 39]. In general, response rates for children were high (above 80% in all countries). Response rates in parents were generally high as well, except in Belgium (62%) and the Netherlands (44%), which may have biased the results, especially because non-response was related to the child’s BMI [32].

### Limitations

Questions on joint parent–child activities (i.e. having breakfast, participating in physical activity/doing sports and watching television) were framed slightly different for children and parents in that children were asked to report on the frequency of these activities they did together with their parents while parents were asked to report the frequency that either both parents or one parent did together with their child. This may partly explain the lower agreement for these practices. Not all constructs could be compared between children and their parents. For instance, no direct corresponding question of whether the parents have rules for each of the five EBRBs (soft drink and fruit juice consumption, having breakfast, taking part in physical activities/doing sports and watching TV) was asked to the parents. Further, no corresponding question was asked to the child on whether the parents negotiate with them about the five EBRBs. Further, we could not compare whether parents gave soft drinks or fruit juices to the child as a reward or to comfort the child and whether parents would prohibit the child to take part in his or her physical activity/sports sessions as a punishment since it was only included in the parent questionnaire.

In this ENERGY sample very few parents with a low education, i.e. less than 7 years, were included. Therefore, our findings on agreement cannot be generalized to parents with less than 7 years of education and their children. Finally, country specific analyses often lacked power and results therefore did not reach statistical significance.

### Implications and future research

Ideally, parental practices should be directly observed in parent -child interactions in their home environment, which of course is unfeasible in large samples due to many reasons including ethical concerns as well as financial and time constraints. A review of studies examining psychometric properties of available parental practice questionnaires would be useful in guiding the design of future questionnaires. Better understanding of the role of parental practices in weight and weight-related behaviours of their children is needed to improve obesity prevention programs. Qualitative research may contribute to understanding the mechanisms’ that link parental practices to EBRB and weight status of children [14] including parental perceptions of EBRBs of their children or the weight status of their child.

## Conclusions

Overall agreement between child and parent reports on parental practices regarding EBRBs was low; with considerable variation across different parental practices. In particular, practices including joint child–parent activities were more often reported differently by child and parent without a clear tendency of either informant. Agreement was lower in less educated and more overweight parents, a subpopulation known to be at risk for raising overweight children. Because we found considerable disagreement we recommend including both parent and children’s perceptions of parental practices in future studies and to take into account the factors that influence disagreement between parent and child perceptions.

## Electronic supplementary material

Additional file 1: Table S5: A list with names and affiliations of the ethical committees in the participating countries that gave their consent to ENERGY. (DOCX 17 KB)

Additional file 2: Table S1: Overview of parental practices used in the child and parent ENERGY questionnaire and their assessed reliability and construct validity. (PDF 122 KB)

Additional file 3: Table S2: Characteristics of parent-child dyads and factors associated with parents not completing a questionnaire for each participating country. (PDF 422 KB)

Additional file 4: Table S3: Percentages of reported parental practices by the child and by the parent, overall and by country. (PDF 375 KB)

Additional file 5: Table S4: Factors associated with the average agreement score (univariate Multilevel linear regression), in total and by country. (PDF 136 KB)

## Abbreviations

EBRB: Energy-balance related behaviour
TV: Television
K: Kappa.
