# Supplementary material for: Agreement between parent and child report on parental practices regarding dietary, physical activity and sedentary behaviours: the ENERGY cross-sectional survey
Source: BMC Public Health. 2014 Sep 5;14:918. doi: 10.1186/1471-2458-14-918 (PMC4169834; doi:10.1186/1471-2458-14-918)
Supplement: Supplementary file 1 — Additional file 1: Table S5: A list with names and affiliations of the ethical committees in the participating countries that gave their consent to ENERGY. (DOCX 17 KB) [file 12889_2013_7052_MOESM1_ESM.docx]

| **Country** | **Name and Affiliation of the Ethical Committee** |
| --- | --- |
| Belgium | The Medical Ethics Committee of the University Hospital Ghent |
| Greece | The Bioethics Committee of Harokopio University |
| Hungary | The Scientific and Ethics Committee of Health Sciences Council |
| The Netherlands | The Medical Ethics Committee of the VU University medical center |
| Norway | The National Committees for Research Ethics in Norway |
| Slovenia | The National Medical Ethics Committee of the Republic of Slovenia |
| Spain | The Clinical Research Ethics Committee of the Government of Aragón |
| Switzerland | Ethics committees of the participating cantons Basel, Bern, Aargau and St. Gallen |

Additional file 1: Table S5
