# Supplementary material for: Agreement between parent and child report on parental practices regarding dietary, physical activity and sedentary behaviours: the ENERGY cross-sectional survey
Source: BMC Public Health. 2014 Sep 5;14:918. doi: 10.1186/1471-2458-14-918 (PMC4169834; doi:10.1186/1471-2458-14-918)
Supplement: Supplementary file 2 — Additional file 2: Table S1: Overview of parental practices used in the child and parent ENERGY questionnaire and their assessed reliability and construct validity. (PDF 122 KB) [file 12889_2013_7052_MOESM2_ESM.pdf]

Supplemental table 1: Overview of parental practices used in the child and parent ENERGY questionnaire and their assessed reliability and construct validity

Child questionnaire	Values	Reliability ICC	Reliability %*	Validity ICC	Validity %†	Parental questionnaire	Values	Reliability ICC	Reliability %*	Validity ICC	Validity %†
Soft drink consumption											
If I ask my parents/ care givers for a fizzy drink of fruit squash, I get one	Always<br>Often<br>Sometimes<br>Not often<br>Never	.62	54	.54	44	If my child asks for soft drinks, I will give it to him/her	Always<br>Often<br>Sometimes<br>Not often<br>Never	.79	75	.43	61
I am allowed to take fizzy drinks or fruit squash whenever I want	Always<br>Often<br>Sometimes<br>Not often<br>Never	.68	52	.30	43	My child is allowed to take soft drinks whenever (s)he wants	Always<br>Often<br>Sometimes<br>Not often<br>Never	.83	73	.64	59
Are there usually fizzy drinks or fruit squash at your home?	Always<br>Often<br>Sometimes<br>Not often<br>Never	.74	61	.52	46	There are soft drinks available at home for my child	Always<br>Often<br>Sometimes<br>Not often<br>Never	.85	73	.67	65
Fruit juice consumption											
I am allowed to take fruit juices whenever I want	Always<br>Often<br>Sometimes<br>Not often<br>Never	.67	59	.48	53	My child is allowed to take fruit juices whenever (s)he wants	Always<br>Often<br>Sometimes<br>Not often<br>Never	.78	67	.59	48
Are there usually fruit juices in your home?	Always<br>Often<br>Sometimes<br>Not often<br>Never	.67	59	.57	52	There are fruit juices available at home for my child	Always<br>Often<br>Sometimes<br>Not often<br>Never	.86	75	.63	61
Having breakfast											
My parents/ care givers encourage me to have breakfast/	I fully agree<br>I agree a bit<br>Neither agree nor disagree<br>I disagree a bit<br>I fully disagree	.65	59	.40	54	I encourage my child to have breakfast	Always<br>Often<br>Sometimes<br>Not often<br>Never	.62	80	.37	43
Are there usually breakfast products (e.g. milk, cereal, bread) at your home?	Always<br>Often<br>Sometimes<br>Not often<br>Never	.42	75	.25	80	There are breakfast products (e.g. milk, cereal, bread) available at home for my child	Always<br>Often<br>Sometimes<br>Not often<br>Never	.68	95	.28	98
How often do you eat breakfast with your parents/care givers?	Never<br>Less than once a week<br>Once a week<br>2-4 days a week<br>5-6 days a week<br>Every day	.74	51	.66	41	How often do you and/or your spouse/partner have breakfast together with your child?	Never<br>Once a week<br>2-4 days a week<br>5-6 days a week<br>Every day	.79	65	.80	71
Physical activity/ sports											
My parents/ care givers encourage me to be physically active/ do sports	I fully agree<br>I agree a bit<br>Neither agree nor disagree<br>I disagree a bit<br>I fully disagree	.65	62	.37	52	I encourage my child to take part in physical activity/sports	Always<br>Often<br>Sometimes<br>Not often<br>Never	.80	78	.30	54

How often do you take part in physical activity/ do sports with your parents/care givers?	Never<br>Less than once a week<br>Once a week<br>2-4 days a week<br>5-6 days a week<br>Every day	.47	47	.24	51	How often do you and/ or your spouse/ partner participate in physical activity/ sports together with your child (e.g. play games outside, ride bikes, walk, play sports together)?	Never<br>Less than once a week<br>Once a week<br>2-4 days a week<br>5-6 days a week<br>Every day	.80	73	.56	57
Watching TV<br>My parents/ care givers allow me to watch television whenever I want/	I fully agree<br>I agree a bit<br>Neither agree nor disagree<br>I disagree a bit<br>I fully disagree	.68	51	.35	33	My child is allowed to watch TV/video/dvd whenever (s)he wants	Always<br>Often<br>Sometimes<br>Not often<br>Never	.72	61	.55	43
If I ask my parents/ care givers to watch television, I can do so	Always<br>Often<br>Sometimes<br>Not often<br>Never	.63	61	.39	57	If my child asks if (s)he is allowed to watch TV/video/dvd, I will allow it	Always<br>Often<br>Sometimes<br>Not often<br>Never	.66	77	.61	58
Do you have a television in your own bedroom?	Yes<br>No	.92	96	.81	91	TV/video/DVD is available in my child's room	Yes<br>No	.97	99	.94	97
How often do you watch television with your parents/care givers?	Never<br>Less than once a week<br>Once a week<br>2-4 days a week<br>5-6 days a week<br>Every day once a day<br>Every day, more than once a day	.64	41	.49	37	How often do you (one parent/spouse/ partner or both) watch television together with your child?	Never<br>Less than once a week<br>Once a week<br>2-4 days a week<br>5-6 days a week	.72	69	.70	69

Abbreviations: ICC, intraclass correlation coefficient

* Percentage agreement between test retest study using the same questionnaire

† Percentage agreement between questionnaire and subsequent interview

From:

Singh AS, Vik FN, Chinapaw MJ, Uijtdewilligen L, Verloigne M, Fernandez-Alvira JM*, et al.* Test-retest reliability and construct validity of the ENERGY-child questionnaire on energy balance-related behaviours and their potential determinants: the ENERGY-project. *Int J Behav Nutr Phys Act*. 2011; 8: 136.

Singh AS, Vik FN, Chinapaw MJ, Uijtdewilligen L, Froydis NV, van Lippevelde W, *et al.* Test-retest reliability and construct validity of the ENERGY-parent questionnaire on parenting practices, energy balance-related behaviours and their potential behavioural determinants: the ENERGY-project. *BMC Res Notes.* 2012; 5: 434.
