# Supplementary material for: Agreement between parent and child report on parental practices regarding dietary, physical activity and sedentary behaviours: the ENERGY cross-sectional survey
Source: BMC Public Health. 2014 Sep 5;14:918. doi: 10.1186/1471-2458-14-918 (PMC4169834; doi:10.1186/1471-2458-14-918)
Supplement: Supplementary file 3 — Additional file 3: Table S2: Characteristics of parent-child dyads and factors associated with parents not completing a questionnaire for each participating country. (PDF 422 KB) [file 12889_2013_7052_MOESM3_ESM.pdf]

Supplemental table 2: Characteristics of parent-child dyads and factors associated with parents not completing a questionnaire for each participating country

		Belgium				Greece				Hungary				Netherlands			
		Participants		Nonres-ponders*		Participants		Nonres-ponders*		Participants		Nonres-ponders*		Participants		Nonres-ponders*	
**Child - Parent dyads (N)**		747		258		992		100		917		98		399		557	
**Child**																	
Gender	Girls	**54.6**		**44.6**		**55.3**		**39.0**		55.7		48.0		51.1		49.9	
	Boys	**45.4**		**55.4**		**44.7**		**61.0**		44.3		52.0		48.9		50.1	
Age (years)	10 <11	26.0		23.6		33.4		29.0		0.7		0.0		19.1		9.7	
	11 <12	47.4		39.9		47.8		41.0		34.5		27.6		46.9		37.7	
	12 <13	25.3		29.8		18.3		15.0		54.9		50.0		**32.3**		**39.3**	
	13 <14	1.2		1.2		0.5		2.0		9.7		20.4		**38.0**		**5.9**	
National language at home	No	**7.2**		**14.7**		**8.5**		**23.0**		2.29		2.0		5.01		9.5	
	Yes	**91.8**		**81.0**		**91.0**		**65.0**		97.6		98.0		93.5		84.4	
Living with	Parents	88.9		85.7		79.2		69.0		77.3		65.3		87.0		78.5	
	Single parent	8.3		7.8		7.7		10.0		**12.4**		**24.5**		11.0		12.8	
	Other adults	2.3		3.1		12.6		10.0		10.1		9.2		**0.0**		**2.7**	
Siblings	No	15.9		16.7		17.2		18.0		21.8		16.3		8.5		7.9	
	Yes	83.5		79.5		82.4		71.0		77.9		81.6		90.0		85.6	
Weight status	Normal	72.7		72.5		54.2		58.0		65.1		64.3		73.2		67.0	
	Overweight†	15.1		14.7		41.5		30.0		24.4		27.6		11.3		17.6	
	Underweight‡	12.1		9.7		3.6		4.0		10.3		8.2		10.5		6.5	
		Mean	SD	Mean	SD	Mean	SD	Mean	SD	Mean	SD	Mean	SD	Mean	SD	Mean	SD
BMI of child	kg/m2	19.0	3.3	18.3	3.1	20.5	3.8	19.8	3.7	19.4	3.6	19.9	3.7	**17.9**	**2.6**	**18.9**	**3.4**
**Parent**																	
Parent	Mother	87.2				81.9				85.3				90.7			
	Father	12.5				17.9				14.4				9.0			
Education	< 14 years	21.8				58.2				51.7				30.3			
	≥ 14 years	77.1				40.3				47.8				68.9			
		Mean	SD			Mean	SD			Mean	SD			Mean	SD		
Age of parent	years	40.9	4.6			41.5	5.9			39.4	5.1			42.4	4.6		
BMI of parent	kg/m2	24.2	4.4			25.2	4.6			24.7	4.6			24.8	4.9		

		Norway				Slovenia				Spain				Switzerland			
		Participants		Nonres-ponders*		Participants		Nonres-ponders*		Participants		Nonres-ponders*		Participants		Nonres-ponders*	
**Child - Parent dyads (N)**		843		161		1010		171		957		64		560		43	
**Child**																	
Gender	Girls	52.9		46.6		**53.3**		**40.9**		51.7		53.1		48.6		39.5	
	Boys	47.1		53.4		**46.7**		**59.1**		48.3		46.9		51.4		50.1	
Age (years)	10 <11	11.7		9.3		31.2		26.3		27.4		21.9		24.1		18.6	
	11 <12	38.7		28.0		47.2		38.0		49.8		42.2		44.3		27.9	
	12 <13	0.0		47.2		21.2		29.2		22.2		29.7		28.0		41.9	
	13 <14	8.3		10.6		0.3		1.2		0.5		3.1		3.4		4.65	
National language at home	No	**3.7**		**11.8**		7.0		11.1		**2.7**		**10.9**		**20.5**		**48.8**	
	Yes	**95.6**		**85.7**		91.9		85.4		**96.9**		**85.9**		**77.3**		**46.5**	
Living with	Parents	87.4		78.3		80.3		76.0		86.0		81.3		81.8		76.7	
	Single parent	**10.2**		**16.2**		**6.9**		**12.9**		5.8		7.8		13.2		18.6	
	Other adults	1.8		2.5		11.9		8.2		8.2		7.8		2.9		0	
Siblings	No	**10.4**		**6.2**		14.6		17.5		16.5		17.2		12.7		9.3	
	Yes	**89.0**		**91.3**		84.2		79.0		83.1		79.7		84.8		86.1	
Weight status	Normal	75.9		75.2		65.2		52.6		69.2		56.3		75.7		62.8	
	Overweight†	13.8		15.5		25.5		28.7		23.9		29.7		13.2		20.9	
	Underweight‡	7.2		8.1		6.8		8.2		5.4		6.3		11.1		9.3	
		Mean	SD	Mean	SD	Mean	SD	Mean	SD	Mean	SD	Mean	SD	Mean	SD	Mean	SD
BMI of child	kg/m2	18.6	2.7	18.7	2.8	19.1	3.3	19.5	3.6	19.1	2.9	19.8	3.2	17.9	2.7	19.1	3.2
**Parent**																	
Parent	Mother	79.1				78.7				80.6				81.6			
	Father	20.3				21.0				19.1				18.2			
Education	< 14 years	35.4				51.5				26.8				69.8			
	≥ 14 years	63.7				47.4				72.4				28.9			
		Mean	SD			Mean	SD			Mean	SD			Mean	SD		
Age of parent	years	42.0	5.0			40.6	4.9			42.6	4.5			42.4	5.7		
BMI of parent	kg/m2	24.6	3.8			24.7	4.2			23.9	3.3			23.6	4.0		

* Coefficient of multilevel (school) logistic regression analyses with response as outcome variable

† Cole TJ, Bellizzi MC, Flegal KM, Dietz WH. Establishing a standard definition for child overweight and obesity worldwide: international survey. *Bmj*. 2000; 320: 1240-3.

‡ Cole TJ, Flegal KM, Nicholls D, Jackson AA. Body mass index cut offs to define thinness in children and adolescents: international survey. *Bmj*. 2007; 335: 194.

Percentages do not add up to 100% due to missing values. In bold statisticyally significant associated with non-response in multilevel (school) model adjusted for child characteristics listed in the table and including BMI as a continous variable
