# Supplementary material for: Agreement between parent and child report on parental practices regarding dietary, physical activity and sedentary behaviours: the ENERGY cross-sectional survey
Source: BMC Public Health. 2014 Sep 5;14:918. doi: 10.1186/1471-2458-14-918 (PMC4169834; doi:10.1186/1471-2458-14-918)
Supplement: Supplementary file 4 — Additional file 4: Table S3: Percentages of reported parental practices by the child and by the parent, overall and by country. (PDF 375 KB) [file 12889_2013_7052_MOESM4_ESM.pdf]

Supplemental table 3: Percentages of reported parental practices by the child and by the parent, overall and by country

Parental Practices	Values	Total C	Total P	Belgium C	Belgium P	Greece C	Greece P	Hungary C	Hungary P	Netherlands C	Netherlands P	Norway C	Norway P	Slovenia C	Slovenia P	Spain C	Spain P	Switzerland C	Switzerland P
Soft drink consumption																			
"If I ask my parents/ care givers for a fizzy drink of fruit squash, I get one" / "If my child asks for soft drinks, I will give it to him/her"	Always or often*	27	20	39	29	21	9	50	29	44	29	10	12	27	28	17	12	19	16
"I am allowed to take fizzy drinks or fruit squash whenever I want" / "My child is allowed to take soft drinks whenever (s)he wants"	Always or often*	25	16	32	23	14	6	43	24	34	19	12	7	33	28	12	9	29	12
"Are there usually fizzy drinks or fruit squash at your home?"/ "There are soft drinks available at home for my child"	Always or often*	43	34	65	50	27	16	56	37	78	53	42	38	27	31	40	40	34	23
Fruit juice consumption																			
"I am allowed to take fruit juices whenever I want" / "My child is allowed to take fruit juices whenever (s)he wants"	Always or often*	62	54	70	51	66	65	58	65	67	37	54	37	66	66	58	57	60	29
"Are there usually fruit juices in your home?"/ "There are fruit juices available at home for my child"	Always or often*	65	65	67	75	73	81	58	58	67	70	56	55	62	57	74	75	55	48
Having breakfast																			
"My parents/ care givers encourage me to have breakfast"/ "I encourage my child to have breakfast"	I fully agree†	49	83	45	84	45	82	68	86	30	86	37	86	63	74	51	90	29	76
Are there usually breakfast products (e.g. milk, cereal, bread) at your home?"/ "There are breakfast products (e.g. milk, cereal, bread) available at home for my child"	Always or often*	92	98	94	98	92	97	92	98	91	99	94	99	88	98	94	99	90	98
"How often do you eat breakfast with your parents/care givers?"/ "How often do you and/or your spouse/partner have breakfast together with your child?"	Never or less than once a week‡	30	27	20	20	47	54	40	35	13	8	20	9	32	31	30	24	21	17
Physical activity/ sports																			
"My parents/ care givers encourage me to be physically active/ do sports"/ "I encourage my child to take part in physical activity/sports"	I fully agree†	63	58	75	57	63	51	75	63	40	58	54	54	83	62	67	61	41	51
"How often do you take part in physical activity/ do sports with your parents/care givers?"/ "How often do you and/or your spouse/partner participate in physical activity/ sports together with your child (e.g. Play games outside, ride bikes, walk, play sports together)?"	Never or less than once a week‡	69	71	75	79	74	80	64	77	70	59	80	71	56	62	58	65	87	72
Watching TV																			
"My parents/ care givers allow me to watch television whenever I want"/ "My child is allowed to watch TV/video/dvd whenever (s)he wants"	I fully agree†	31	20	41	25	24	21	46	26	39	19	24	33	36	14	22	10	11	6
"If I ask my parents/ care givers to watch television, I can do so" / "If my child asks if (s)he is allowed to watch TV/video/dvd, I will allow it"	Always or often*	50	43	53	44	52	46	74	66	45	47	55	55	50	36	30	21	27	23
"Do you have a television in your own bedroom?"/ "TV/video/DVD is available in my child's room?"‖	Yes§	35	32	25	24	42	40	65	61	39	33	39	37	24	22	28	20	15	14

"How often do you watch television with your parents/care givers?"/ "How often do you (one parent/spouse/ partner or both) watch television together with your child?"	Never or less than once a week‡	32	33	18	29	34	35	29	34	30	23	32	18	34	44	29	24	53	60

Abbreviations: C, Child; P, Parent

*versus: Sometimes, not often, never

†versus: I agree a bit, neither disagree nor agree, I disagree a bit, I fully disagree

‡ versus: Once a week, 2-4 days a week, 5-6 days a week, every day

§versus: No

Percentages are presented and cells of values are coloured in light grey if the proportion (reported by the child) differed by more than 15% of the average proportion (reported by the child) over all countries in either direction
