# Supplementary material for: Agreement between parent and child report on parental practices regarding dietary, physical activity and sedentary behaviours: the ENERGY cross-sectional survey
Source: BMC Public Health. 2014 Sep 5;14:918. doi: 10.1186/1471-2458-14-918 (PMC4169834; doi:10.1186/1471-2458-14-918)
Supplement: Supplementary file 5 — Additional file 5: Table S4: Factors associated with the average agreement score (univariate Multilevel linear regression), in total and by country. (PDF 136 KB) [file 12889_2013_7052_MOESM5_ESM.pdf]

Supplemental table 4: Factors associated with the average agreement score (univariate multilevel linear regression), in total and by country

		Total			Belgium			Greece			Hungary			Netherlands			Norway			Slovenia			Spain			Switzerland		
		b*	95%CI		b†	95%CI		b†	95%CI		b†	95%CI		b†	95%CI		b†	95%CI		b†	95%CI		b†	95%CI		b†	95%CI	
Child characteristics																												
**Gender**																												
	Boy	**-1.82**	**-2.53**	**-1.10**	**-2.33**	**-4.40**	**-0.25**	-1.44	-3.22	0.35	-1.31	-3.20	0.58	-1.46	-4.14	1.23	**-3.00**	**-4.92**	**-1.08**	**-1.62**	**-3.54**	**0.29**	**-1.80**	**-3.65**	**0.05**	-1.44	**-3.85**	**0.97**
**Age**	10 years																											
	11 years	0.55	-0.39	1.49	0.39	-2.14	2.92	-1.40	-3.39	0.60	1.01	-10.68	12.70	-0.21	-3.88	3.47	**4.06**	**0.85**	**7.26**	**2.05**	**-0.14**	**4.24**	1.35	-0.83	3.54	-1.96	-5.05	1.12
	12 years	**1.41**	**0.35**	**2.46**	0.31	-2.59	3.21	0.85	-1.74	3.43	1.52	-10.13	13.18	1.71	-2.17	5.60	**4.50**	**1.32**	**7.68**	**0.76**	**-1.91**	**3.43**	3.71	1.08	6.34	-0.10	-3.53	3.33
	13 years	**3.01**	**0.78**	**5.23**	8.53	-1.07	18.13	n.a.			3.15	-8.82	15.13	-6.77	-18.81	5.27	**6.43**	**2.06**	**10.79**	-16.07	-37.14	4.99	2.24	-14.08	18.55	-1.19	-8.14	5.76
**Weight status**																												
	Normal																											
	Overweight	**-1.28**	**-2.15**	**-0.41**	-0.85	-3.80	2.11	-0.68	-2.51	1.15	**-2.26**	**-4.48**	**-0.04**	-1.67	-5.85	2.50	-0.74	-3.52	2.05	**-2.22**	**-4.43**	**-0.02**	-0.82	-2.99	1.35	-1.19	-4.79	2.40
	Underweight	-0.22	-1.55	1.11	-0.21	-3.42	3.01	0.28	-4.51	5.08	-0.53	-3.67	2.62	2.13	-2.17	6.44	-3.04	-6.72	0.64	0.72	-3.04	4.49	-1.91	-5.97	2.15	1.68	-2.15	5.51
Parental characteristics																												
Parent																												
	Father	-0.07	-1.01	0.88	0.59	-2.55	3.74	1.88	-0.44	4.20	-0.46	-3.14	2.21	2.49	-2.19	7.17	-1.47	-3.85	0.91	-1.85	-3.92	0.46	1.24	-1.11	3.59	-1.40	-4.50	1.70
**Age**	per year	**0.12**	**0.05**	**0.19**	0.18	-0.04	0.41	0.14	-0.02	0.29	0.00	-0.18	0.19	0.22	-0.08	0.51	**0.19**	**0.00**	**0.38**	0.20	-0.11	0.29	**0.13**	**-0.08**	**0.34**	0.12	-0.09	0.34
BMI‡	per kg/m2	0.00	0.00	0.00	-0.11	-0.35	0.14	0.00	0.00	0.00	-0.07	-0.28	0.14	**-0.36**	**-0.65**	**-0.08**	-0.13	-0.40	0.14	**-0.35**	**-0.59**	**-0.12**	-0.2177	-0.5	0.07	0.00	0.00	0.00
**Education**																												
	<14 yrs																											
	≥ 14 yrs	**2.41**	**1.66**	**3.17**	1.54	-0.96	4.05	2.39	0.57	4.20	1.19	-0.72	3.10	0.23	-2.72	3.19	**4.03**	**2.02**	**6.04**	**4.85**	**2.92**	**6.79**	1.72	-0.38	3.82	0.48	-2.16	3.12
Employment																												
	No	-0.94	-1.99	0.10	-0.65	-4.43	3.12	-1.70	-4.03	0.62	-0.66	-3.22	1.90	2.63	-0.68	5.93	-2.03	-5.93	1.87	-2.32	-5.84	1.21	-2.27	-4.69	0.16	1.77	-1.22	4.75
**Perception of the child's weight**																												
	Normal																											
	Overweight	**-1.18**	**-2.07**	**-0.30**	1.61	-1.13	4.36	-0.30	-2.21	1.60	**-2.84**	**-5.08**	**-0.59**	1.34	-2.42	5.11	-1.76	-4.76	1.24	-2.68	-4.93	-0.44	-2.12	-4.42	0.18	0.28	-3.09	3.65
	Underweight	**-1.40**	**-2.55**	**-0.25**	-0.24	-3.63	3.14	-0.23	-3.35	2.90	**-4.07**	**-6.95**	**-1.18**	-2.24	-6.60	2.12	**-3.39**	**-6.64**	**-0.14**	-0.89	-4.12	2.33	-1.66	-4.35	1.02	2.77	-1.03	6.56
Family characteristics																												
Native language at home																												
	Yes	0.42	-1.05	1.89	1.15	-2.90	5.19	-1.55	-4.79	1.68	0.75	3.21	0.23	-5.70	-11.66	0.26	2.30	-2.91	7.52	**5.24**	**1.55**	**8.93**	-4.08	-9.78	1.63	-0.38	-3.39	2.63
Siblings																												
	Yes	0.20	-0.78	1.19	2.19	-0.63	5.02	0.31	-2.04	2.66	-1.22	-3.49	1.04	2.59	-2.29	7.47	-0.14	-3.28	3.00	0.42	-2.26	3.11	0.30	-2.18	2.79	-0.80	-4.41	2.81
Living with																												
	Both parents																											
	Mother or Father	-1.22	-2.47	0.03	-0.27	-4.03	3.49	-2.45	-5.79	0.90	-0.48	-3.34	2.39	-2.55	-6.77	1.67	1.56	-1.67	4.80	-4.08	-7.82	-0.34	-2.41	-6.48	1.65	0.28	-3.09	3.65
	Other	-1.15	-2.55	0.25	-6.62	-13.52	0.29	-1.01	-3.71	1.69	-2.06	-5.19	1.08	-1.68	-20.15	16.78	-0.39	-8.10	7.31	-0.47	-3.45	2.51	0.66	-2.71	4.03	2.77	-1.03	6.56

Abbreviations: BMI, body mass index; CI, confidence interval

* Univariate multilevel linear regression model (country, school) with agreement score as outcome

† Univariate multilevel linear regression model (school) with agreement score as outcome

‡ Adjusted for parent's gender

Numbers in bold represent statistically significant associations with the overall agreement score (P <0.05)
